# Quality improvement of oil extracted from flaxseeds (*Linum usitatissimum* L.) incorporated with olive leaves by cold press

**DOI:** 10.1002/fsn3.4044

**Published:** 2024-02-20

**Authors:** Ramin Teimouri Okhchlar, Afshin Javadi, Sodeif Azadmard‐Damirchi, Mohammadali Torbati

**Affiliations:** ^1^ Department of Food Science and Technology, Mamaghan Branch Islamic Azad University Mamaghan Iran; ^2^ Department of Food Hygiene, Faculty of Veterinary, Tabriz Medical Science Islamic Azad University Tabriz Iran; ^3^ Department of Food Science and Technology, Faculty of Agriculture University of Tabriz Tabriz Iran; ^4^ Department of Food Science and Technology, Faculty of Nutrition and Food Science Tabriz University of Medical Sciences Tabriz Iran

**Keywords:** fatty acid, flaxseed, olive leaf, oxidation, shelf life

## Abstract

Flaxseed oil has a high amount of α‐linolenic acid (an ω3 essential fatty acid), but it is very prone to oxidation. Therefore, olive leaves were used as a rich source of phenolic compounds with flaxseeds upon oil extraction by cold press to enhance the oxidative stability of extracted oils. Oil from flaxseeds with unblanched leaves and blanched leaves at level of (0 [control sample], 2.5, 5, 7.5, and 10% w/w) was extracted by cold press. Quality of extracted oils was evaluated for 90 days of storage at room condition. Incorporation of unblanched olive leaves could increase the acid value of the extracted oils up to 2.0 (mg KOH/g oil) compared to the other samples. Oxidation of the flaxseed oil could be delayed by the addition of blanched olive leaves up to 5%. Oil extracted from flaxseeds incorporated with blanched olive leaves had higher content of carotenoids (up to 33.7 mg/kg oil), chlorophylls (up to 35.7 mg/kg oil), and phenolic compounds (up to 200 mg/kg oil). Also, oxidative stability of extracted oils was higher up to 7.5% of blanched olive leaves (11.4 h) compared to control sample (7.2 h) and other oil samples. Polyunsaturated fatty acids of the oil samples were well preserved by the incorporation of blanched olive leaves. Based on the obtained results, incorporation of suitable amount of blanched olive leaves (up to 7.5%) with flaxseeds before oil extraction by press can be an appropriate procedure to produce oils with high content of bioactive components and suitable oxidative stability.

## Graphical abstract

Flaxseed oil is a rich source of α‐linolenic acid, but it is very sensitive to oxidation, therefore, it is necessary to use suitable methods to enhance its stability. Olive leaves as a rich source of antioxidants were used with flaxseeds upon oil extraction by cold press. Incorporation of suitable amount of blanched olive leaves (up to 7.5%) with flaxseeds before oil extraction can be an appropriate procedure to extract oils with high content of bioactive components and good oxidative stability.

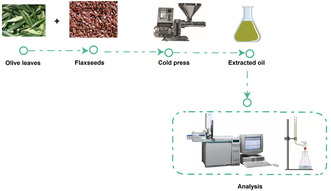

## 1 INTRODUCTION

Flaxseed (*Linum usitatissimum* L.) has a high content of oil (40%–50%) rich in linolenic acid (up to 70%) as an essential ω3 fatty acid, which makes it a beneficial oil from the nutritional point of view (Goyal et al., 2015). Many published scientific reports have proven the positive effect of flaxseed oil on the reduction and prevention of many different diseases (Wang et al., 2017). However, this oil has poor oxidative stability due to its fatty acid composition and high content of polyunsaturated fatty acids, which creates limitations in its uses as a conventional oil in the market and daily uses (Hashempour‐Baltork et al., 2018; Mikołajczak & Tańska, 2022).

There are different approaches to increase the oxidative stability of the edible oils, such as mixing with stable oils, hydrogenation, and using antioxidants. Blending is used for many purposes; however, this method can alter the fatty acid composition and lower the essential ω3 fatty acid level of oils such as flaxseed oil, and also, there is a possibility of phase separation during storage (Hashempour‐Baltork et al., 2016). Hydrogenation is not suitable from a nutritional point of view as *trans* isomers of fatty acids are formed, which trigger different diseases and there are many recommendations to lower its intake (Hashempour‐Baltork et al., 2016; Kummerow, 2009).

Using antioxidants to enhance vegetable oil's oxidative stability is a common way. Antioxidants can be natural or synthetic. However, there is a growing trend to use natural antioxidants. Plants are a rich source of different antioxidative components, which are used to reduce vegetable oils' oxidation. Olive (*Olea europaea*) leaves are one of the rich sources of antioxidative components called phenolic compounds among plant sources (Krichene et al., 2015; Mikołajczak & Tańska, 2022). Olive leaves are available as waste and inexpensive byproduct of olive fruit production throughout the year (Krichene et al., 2015).

Enzymes, such as lipases and lipoxygenases, can lower oil stability via hydrolysis and oxidation, respectively (Mazaheri et al., 2019b). Olive leaves can have different amounts of these enzymes (Sofo et al., 2004), which is why it is advisable to inactivate them before application as antioxidant sources. These enzymes can be inactivated via blanching of the plant sources (Ayele et al., 2022).

There have been many reports on the application of olive leaf extracts as antioxidants in vegetable oils and food formulations (Dauber et al., 2022; Kiritsakis et al., 2017; Şahin et al., 2017). The application and using plant extracts have many advantages in the food industry. However, they need extraction and also, in many cases, concentration, drying steps, and limitation of solubility of extracts in vegetable oils (Dauber et al., 2022). Olive leaves have been used with sunflower, chia, and sesame seeds in the simultaneous extraction of oil by pressurized propane method, which indicated that this method can reduce lipid oxidation and improve the bioactive components and nutritional characteristics of the extracted oils (Jaski et al., 2022, 2023).

Using plant source with oilseeds while oil extraction by the cold press would eliminate the plant extract preparation and applying steps and limitations. Due to the poor stability of flaxseed oil and the high content of antioxidative components of olive leaves, the aim of this study was to introduce a new method of oil extraction by cold press from flaxseeds incorporated with olive leaves. Also, blanching was used to inactivate the olive leaves enzymes to investigate its effect on the extracted oil stability and quality.

## 2 MATERIALS AND METHODS

### 2.1 Materials

Flaxseeds were bought from a local market (Tabriz, Iran). Olive leaves (*O. europaea* L.) were collected from a local olive garden (Rudbar, Iran) during fruit harvesting (October 2020). All other chemical materials were from Sigma‐Aldrich Co. (St. Louis, Missouri, United States).

### 2.2 Methods

#### 2.2.1 Olive leaves blanching

Olive leaves without any possible impurities were subjected to thermal treatments of 90°C distilled water for 1 min, which was examined to pass the blanching test (Beveridge & Weintraub, 1995).

#### 2.2.2 Extraction process

Flaxseed oil was coextracted with blanched and unblanched olive leaves at the levels of (0 [control sample], 2.5, 5, 7.5, and 10% [w/w]) using a press (model P500R, Anton Fries, Germany) (Piravi‐vanak et al., 2022). The temperature of extracted oil was kept under 40°C during the oil extraction.

#### 2.2.3 Storage conditions

The oil samples were held in dark bottles at room condition (20–25°C) in a dark place for 90 days. Oil samples were analyzed in the extraction day (day 1) and each month during storage.

#### 2.2.4 Determination of acid value (AV)

The AV was determined following the AOAC method using the titration of an oil sample dissolved in ethanol:chloroform by KOH (AOAC, 1999).

#### 2.2.5 Determination of peroxide value (PV)

The PV was measured following the AOAC method (AOAC, 1999). Oil (5 g) was dissolved in a mixture of chloroform:acetic acid, and then potassium iodide (KI) solution was added. After keeping in the dark for 1 min, water and starch solution were added, and the mixture was titrated by sodium thiosulfate. PV was expressed in mg oxygen equivalent per kg of oil (meqO_2_/kg oil).

#### 2.2.6 Determination of total phenol content

For determination of total phenol content, extracted oil samples were dissolved in the *n*‐hexane and then mixed with methanol–water (80:10 v/v), according to the method described by Herchi et al. (2011). Then, the prepared methanolic extract (0.2 mL) was mixed with diluted Ciocalteu reagent (1:10, v/v) (1 mL) and sodium carbonate 7% (3 mL). Then, the obtained mixtures were kept at room condition for 30 min in the dark place. The absorbance of the prepared mixtures was measured using a ultraviolet–visible (UV–Vis) spectrophotometer at 760 nm. Also, the reference sample was pure ethanol, and the standard compound was gallic acid at the concentration of 0–0.06 mg/mL. Also, the total phenol content of the oil samples was expressed as milligram gallic acid equivalents (GAEs)/kg oil.

#### 2.2.7 Determination of the chlorophyll content

For determination of the chlorophyll content (as mg of pheophytin/kg of oil), the absorbance of the oil samples dissolved in cyclohexane was determined at 670 nm using the method of Jaber et al. (2012).

#### 2.2.8 Carotenoid contents

For determination of the carotenoid content (as mg/kg of oil), the absorbance of the oil samples dissolved in cyclohexane was determined at 470 nm following the method described by Şahin et al. (2017).

#### 2.2.9 Fatty acid profile

Fatty acid methyl esters (FAMEs) were prepared from oil samples by following the method described by Fathi‐Achachlouei et al. (2019). For this purpose, oil sample (0.1 g) was dissolved in 5 mL *n*‐hexane, and then 1 mL potassium hydroxide (1 M) was added and shaken at 50°C for 20 min. Then, the upper phase containing FAME was separated for further analysis. Separation and analysis of FAME were performed by gas chromatography (GC) (7890A, Agilent, USA) equipped with capillary column (BPX70: 50 m × 0.22 mm, 0.25 μm film, Agilent Technologies). The carrier gas was helium at a constant flow rate of 1.0 mL/min. The temperature of the flame ionization detector was 230°C. The temperature program used for analysis was as follows: 158°C (5 min) and then increased by the rate of 2°C/min to 220°C. Retention times of the pure standards were used to determine the fatty acid peaks. The obtained peak areas from GC were used to calculate the percentage of each fatty acid from the total fatty acid content.

#### 2.2.10 Rancimat analysis

Rancimat apparatus (Metrohm 743; Metrohm Co., Herisau, Switzerland) was used to determine the oxidative stability (as induction period expressed as hour) of oil samples following the Association of Official Analytical Chemists (AOAC) method (AOAC, 1999). The oxidation process was conducted on oil samples (2.5 g) at 110°C and air velocity of 20 L/h.

### 2.3 Statistical analysis

All the experiments were conducted in triplicate. One‐way analysis of variance (ANOVA) was used for the statistical analysis. Also, Duncan's test at the probable level of 5% (*p* < .05) was used for the mean difference determination between the obtained data. Performing Pearson's correlation between the obtained oxidative stability and other properties of the extracted oils was done by Minitab 14 (Minitab Inc., PA, USA).

## 3 RESULTS AND DISCUSSION

Free fatty acids (FFAs) can be formed by hydrolysis of triacylglycerols caused by heat or enzymes in the presence of water. A high content of FFA is quality concern in the fats and oils, as they can lower the smoking point and also decrease the oxidative stability of the product. Therefore, there are many researches to control and prevent the formation of FFA. The FFA level can be presented as an acid value, but there is a limit for this value (4 mg KOH/g oil) in the Codex Alimentarius for virgin and cold press oils (Codex, 1999).

**FIGURE 1:**
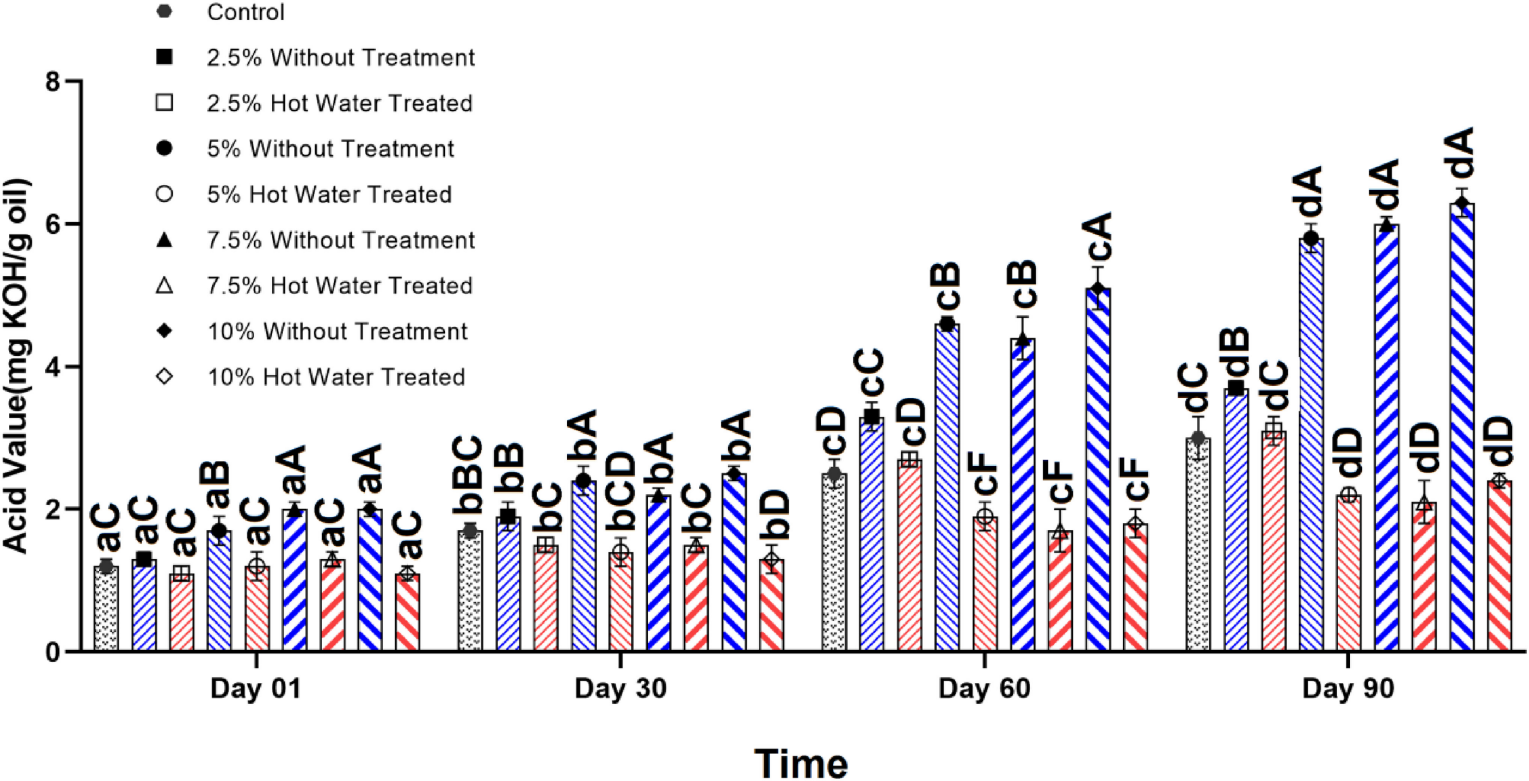
Changes in the acid value (mg KOH/g oil) of oil extracted by cold press from flaxseeds incorporated with different levels (%) of blanched and unblanched olive leaves during storage. Different small and capital letters indicate significant differences between days and treatments, respectively.

All the extracted oil samples had the AV of less than 4 (mg KOH/g oil) on the production day, but this value increased to higher than the maximum limit in the oil samples extracted from the flaxseeds incorporated with the unblanched olive leaves at a level of higher than 5% after 60 days of storage (Figure 1). In all oil samples, AV was increased during the storage period from 1.1 to 6.3 (mg KOH/g oil). However, oil extracted from the flaxseeds incorporated with blanched olive leaves had the lowest AV even after 90 days of storage, and AV was lower than the maximum level (Figure 1). These results show that using blanched olive leaves can delay the increase of AV by interrupting the enzyme activity.

There are many reports showing the increase of FFA and as a result increase of AV during storage due to the hydrolysis of triacylglycerols. This could be delayed by the storage of the fats and oils at the low temperature and also control of water activity (Naebi et al., 2022). The results of this study showed that using the blanched olive leaves could be the new alternative in controlling the hydrolysis reaction.

**FIGURE 2:**
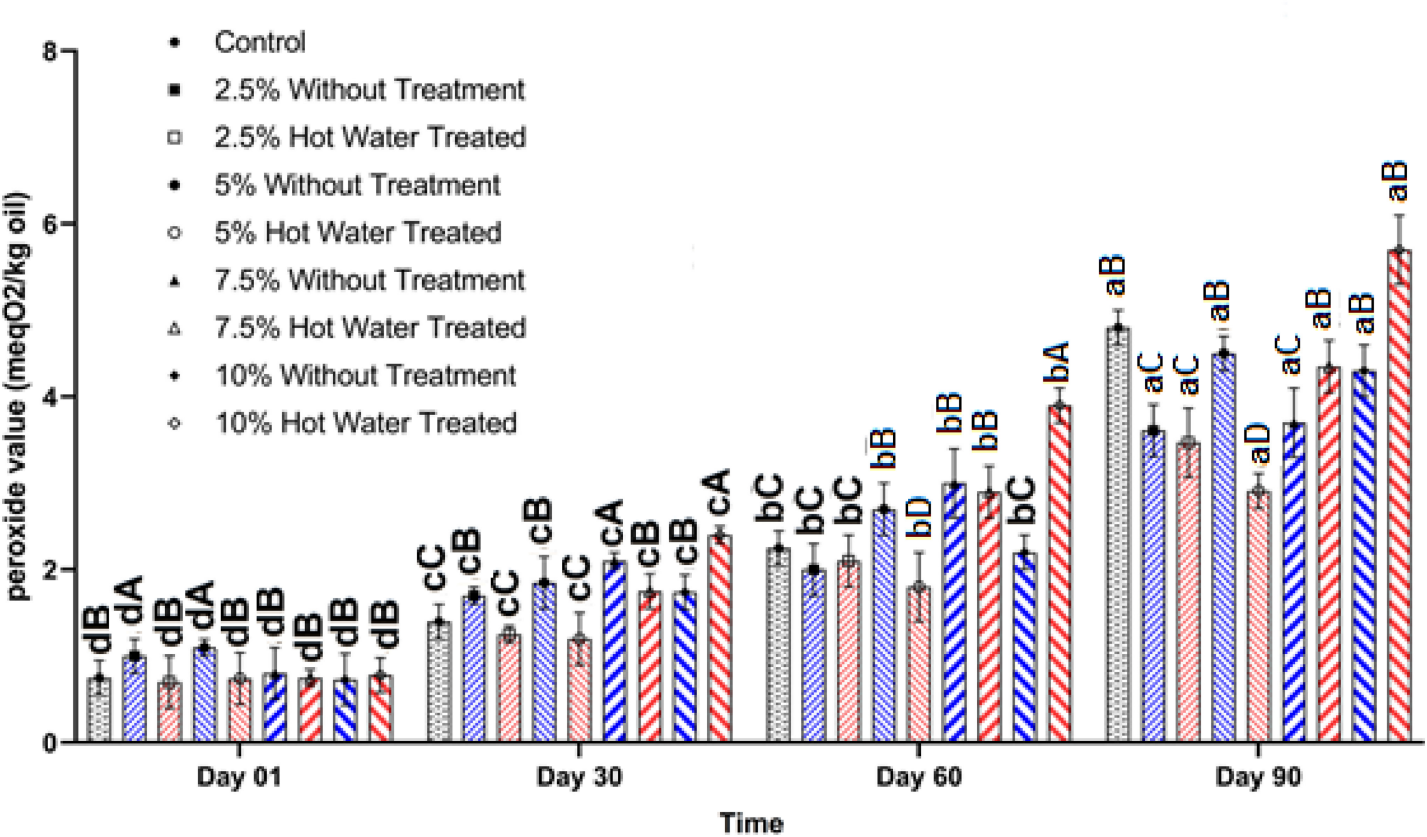
Changes in the peroxide value (meqO_2_/kg oil) of oil extracted by cold press from flaxseeds incorporated with different levels (%) of blanched and unblanched olive leaves during storage. Different small and capital letters indicate significant differences between days and treatments, respectively.

Hydroperoxides or peroxides are the primary oxidation products that can be produced by oxidation. Oxidation of fats and oils can be done in three major different ways, namely autooxidation, photooxidation, and enzymatic oxidation. Autoxidation is a usual way in all fats and oils, but for photooxidation and enzymatic oxidation, there is a need for sensitizers, light, and enzymes, respectively. Chlorophyll is the main sensitizer in vegetable oils such as flaxseed oil (Mikołajczak & Tańska, 2022). High peroxide value is unacceptable in the vegetable oils, as there is a limit for PV in the different standards such as Codex Alimentarius of not more than 15 (meqO_2_/kg oil) for virgin and cold press oils (Codex, 1999). Based on the obtained results, all the produced oil samples were within the Codex Alimentarius limit even after 90 days of storage. Oil extracted from the flaxseeds incorporated with olive leaves without any treatment by the 5% level could increase the PV on the extraction day (Figure 2). The PV of all oil samples was raised during the storage. However, the lowest PV (2.9 meqO_2_/kg oil) belonged to the oil samples extracted from the flaxseed with 5% of blanched olive leaves. However, blanched olive leaves at a level of higher than 7.5% could have a peroxidative effect and increase the PV (Figure 2). Therefore, it is advisable to use the blanched olive leaves with oilseeds in a suitable amount, which was 5% for the flaxseed. There have been many reports on the antioxidative effect of herbs rich in phenolic compounds in fats and oils (Dauber et al., 2022; Mikołajczak et al., 2021). Also, it was demonstrated that higher phenolic compounds could have an adverse impact on the stability of vegetable oils, which was in agreement with the obtained results in this study.

Phenolic compounds are one of the most important minor components present in vegetable oils, which are responsible for their taste, nutritional value, and oxidative stability. Olive oil is one of the leading oils among vegetable oils, which has a high content of phenolic compounds (Kiritsakis et al., 2017). Olive leaves are high in phenolic compounds as well, which makes them suitable and rich source of these compounds to be used for the fortification and valorization of other food items. Olive leaves extract has been used in many researches, and results have shown their antioxidative and favorable health properties (Dauber et al., 2022; Kiritsakis et al., 2017; Şahin et al., 2017). The extraction of olive leaves antioxidative components has several steps, and simplification of these steps can be essential from economic and technological points of view. One way is using the leaves with oilseeds upon oil extraction by screw press. During pressing, olive leaves are squeezed and mixed harshly with the oil phase and their bioactive components are leached and extracted to the oil medium.

**FIGURE 3:**
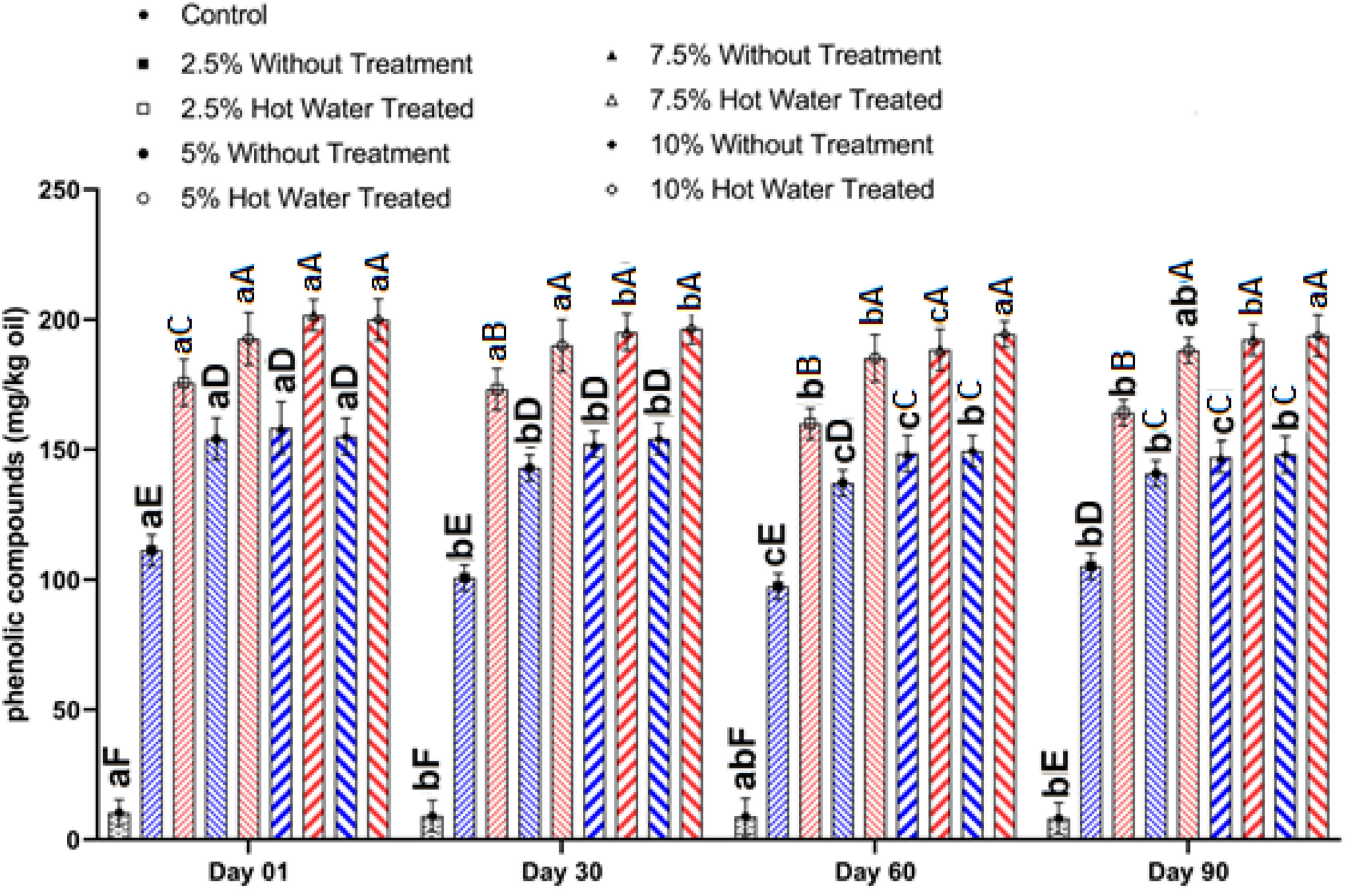
Changes in the phenolic compounds (mg/kg) of oil extracted by cold press from flaxseeds incorporated with different levels (%) of blanched and unblanched olive leaves during storage. Different small and capital letters indicate significant differences between days and treatments, respectively.

Extracted oil from flaxseeds had 10 (mg GAE/kg oil) total phenol content, which was increased several times (10–20 times) by extraction of oils from the flaxseeds with different percentages of olive leaves (Figure 3). The obtained results showed that the incorporation of leaves blanched with hot water could increase the phenolic compounds at a higher level in the extracted oils compared with the untreated leaves (Figure 3). This can be due to the cell membrane rupture and softening effect of blanching by hot water, which allows and facilitates the higher percolation of the phenolic compounds from leaves into the oil phase.

Total phenol contents were increased during the 30 days of storage, and then their contents were decreased. The reason for the increase in the early stage of the storage can be due to the decomposition of complex phenolic components to the simpler units, which could be detected and measured by the spectrophotometric methods (Naebi et al., 2022; Sofo et al., 2004). However, the overall trends were the reduction of the phenolic compounds during the 90 days of storage and reduction of around 10% of the total phenol content. The decline in the phenolic content can be due to several reasons, such as oxidation and decomposition. There are several reports on the changes of the phenolic components in vegetable oils during storage. Generally, the obtained results were in agreement with the previously reported data (Kiritsakis et al., 2017).

The obtained results showed that oil extraction from flaxseeds with olive leaves could increase the phenolic compounds of the oils, which are important from nutritional and technological points of view. These results are in agreement with the previously published data. It has been reported that simultaneous oil extraction from sunflower with olive leaves using solvents could increase the bioactive components of extracted oils (Jaski et al., 2022, 2023). Also, oil extracted from pomegranate seeds with green tea leaves by cold press had higher total phenolic compounds (Dezashibi et al., 2022). This procedure can make vegetable oils more functional with positive health effects. It can make sensitive vegetable oils such as flaxseed oil more stable against oxidation, which is in agreement and approved with the PV and rancimat results.

Carotenoids in a suitable amount have positive effects on the oxidative stability of vegetable oils and also give a fair yellow color. They are also precursors of vitamin A and enhance nutritional properties of the vegetable oils. Flaxseed oil has 15.4 (mg/kg oil) carotenoids that are comparable with many vegetable oils and lower than palm oil (Mannucci et al., 2019). Carotenoids can quench the singlet oxygen and can also be reduced to some extent during storage via decomposition and oxidation. Results showed that carotenoids were reduced by 10%–30% during the 90 days of storage (Table 1).

**TABLE 1:** Changes in carotenoid content (mg/kg oil) of oil extracted by cold press from flaxseeds containing different levels of blanched olive leaves during storage.

Oil*	Time (day)			
	1	30	60	90
Control	15.4 ± 0.1^aG^ **	14.8 ± 0.1^bF^	13.2 ± 0.2^bF^	12.0 ± 0.3^cF^
Leaves without treatment				
2.5%	20.1 ± 0.1^aF^ ***	18.7 ± 0.2^bE^	15.0 ± 0.2^cE^	14.2 ± 0.1^dE^
5%	22.3 ± 0.2^aE^	20.4 ± 0.2^bD^	18.4 ± 0.1^cD^	16.8 ± 0.4^dD^
7.5%	25.6 ± 0.5^aCD^	23.7 ± 0.1^bBC^	20.8 ± 0.3^cC^	19.0 ± 0.1^dC^
10%	26.0 ± 0.1^aC^	24.0 ± 0.4^bB^	22.5 ± 0.3^cBC^	21.7 ± 0.2^cB^
Leaves treated with hot water				
2.5%	24.1 ± 0.1^aD^	22.0 ± 0.1^bC^	20.5 ± 0.1^cC^	18.9 ± 0.2^dC^
5%	26.3 ± 0.2^aC^	25.1 ± 0.2^bB^	24.0 ± 0.2^bB^	23.1 ± 0.1^cB^
7.5%	28.9 ± 0.5^aB^	24.3 ± 0.1^bB^	23.8 ± 0.3^bB^	22.9 ± 0.3^cB^
10%	33.7 ± 0.1^aA^	29.7 ± 0.2^bA^	28.2 ± 0.2^cA^	26.5 ± 0.5^dA^

* Oil samples extracted from flaxseeds with different levels of olive leaves.

** Data are mean ± standard deviation.

*** Different small letters in each row indicate a significant difference between days. Different capital letters in each column indicate significant differences for treatments.

Extraction of oil from a blend of flaxseeds with olive leaves could increase the carotenoid content of the extracted oil (Table 1). This can be explained by the high content of carotenoid in the olive leaves. Also, flaxseed oil is a suitable medium and can act as a solvent for carotenoids. Also, olive leaves higher than 7.5% were not effective in the increase of carotenoid content in the extracted oil.

Pretreatment of leaves with hot water could affect the carotenoid extraction to the flaxseed oil as the texture of the leaves becomes soft and more prone to the pressure made by the screw press. The carotenoid content of the oil samples extracted from the flaxseeds with hot water‐treated olive leaves was 10%–15% higher than the oil samples extracted from the flaxseeds with untreated olive leaves (Table 1).

Chlorophyll is responsible for the photooxidation of vegetable oils in the presence of light, but it can act as an antioxidant in the dark condition. Flaxseed oil had lower content (1.2 mg/kg oil) of chlorophyll compared with other vegetable oils such as olive oil, canola oil, soybean oil, and black cumin seed oil (Bianchi et al., 2015; Endo et al., 1992; Giuliani et al., 2011; Sofo et al., 2004). Olive leaves are rich sources of chlorophyll; therefore, incorporating them with flaxseeds upon oil extraction by the press could increase the chlorophyll content of extracted oils (Table 2). This increase was higher in the oil extracted from seeds with leaves treated with hot water as their texture was softer and more prone to rupturing. Chlorophyll is an oil‐soluble pigment, and it can be easily leached into the oil. However, oil with high content of chlorophyll should be kept in the dark conditions or in dark containers to avoid photooxidation. A higher level of leaves could impair the visual quality of the extracted oils as it gives a very green color; therefore, it is advisable to avoid using the higher level of leaves from the stability and consumer acceptability points of view.

**TABLE 2:** Changes in chlorophyll content (mg/kg oil) of oil extracted by cold press from flaxseeds containing different levels of blanched olive leaves during storage.

Oil*	Time (day)			
	1	30	60	90
Control	1.19 ± 0.3^aH^ **	1.14 ± 0.1^aI^	0.91 ± 0.4^aH^	0.75 ± 0.3^bH^
Leaves without treatment				
2.5%	14.17 ± 0.1^aG^ ***	11.20 ± 0.2^bH^	10.13 ± 0.2^cG^	8.70 ± 0.1^dG^
5%	19.60 ± 0.2^aE^	15.0 ± 0.2 ^bG^	14.1 ± 0.5^cF^	12.35 ± 0.2^dF^
7.5%	22.88 ± 0.4^aD^	18.9 ± 0.1 ^bE^	16.3 ± 0.3^cE^	15.2 ± 0.1^cE^
10%	28.4 ± 0.1^aC^	20.3 ± 0.3^bD^	19.56 ± 0.3^cD^	17.85 ± 0.4^cD^
Leaves treated with hot water				
2.5%	17.9 ± 0.1 ^aF^	17.3 ± 0.1^bF^	14.2 ± 0.1^cF^	13.9 ± 0.2^cF^
5%	28.9 ± 0.2^aC^	25.6 ± 0.2^bC^	24.7 ± 0.2^cC^	23.0 ± 0.5^cC^
7.5%	33.2 ± 0.3^aB^	29.4 ± 0.1^bB^	26.3 ± 0.3^cB^	25.2 ± 0.3^dB^
10%	35.7 ± 0.1^aA^	33.0 ± 0.2^bA^	31.2 ± 0.1^cA^	30.1 ± 0.1^dA^

* Oil samples extracted from flaxseeds with different levels of olive leaves.

** Data are mean ± standard deviation.

*** Different small letters in each row indicate a significant difference between days. Different capital letters in each column indicate significant differences for treatments.

Chlorophyll was decomposed and reduced at a level of 10%–20% during the storage of oil samples in the room condition (Table 2). Lower and higher contents of chlorophyll after 90 days of storage belonged to the control sample and the oil extracted from seeds with 10% of blanched leaves, respectively. There are scientific reports that this pigment can be decomposed and reduced during the storage of vegetable oils (Caponio et al., 2005; Mannucci et al., 2019; Mazaheri et al., 2019a).

**FIGURE 4:**
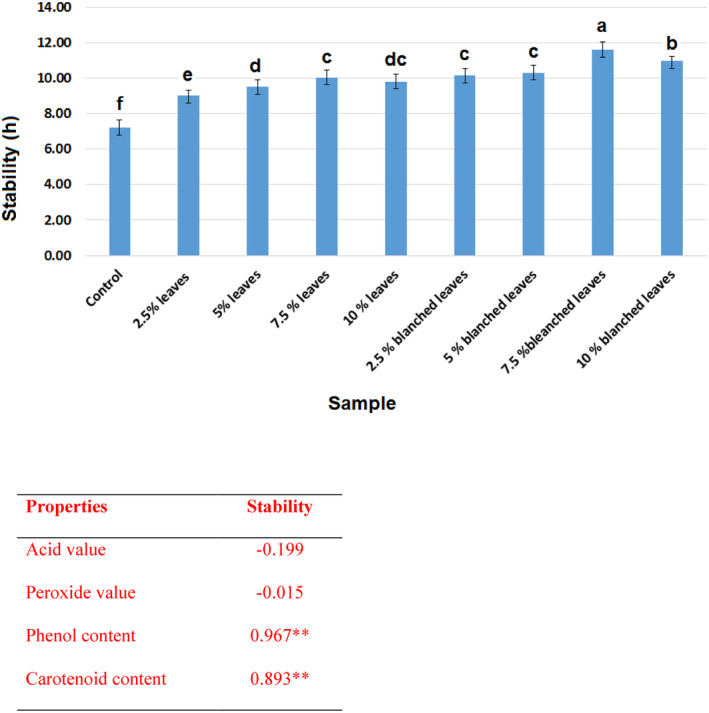
The oxidative stability (hour) of oil extracted by cold press from flaxseeds with different levels of blanched and unblanched olive leaves. Different small letters indicate significant differences in oil samples. **p < .01. LSD, least significant difference.

Accelerated oxidation test methods can give rapid results on the oil stability and can be a prediction on the condition and stability during a long period of storage. Using rancimat to determine the induction period of the vegetable oils can be helpful, and it is possible to compare the effects of any pretreatments on the produced oils. Results showed that the control sample had the lowest oxidative stability (7.2 h), but the oil extracted from seeds with 7.5% of blanched leaves had the highest stability (11.4 h) (Figure 4), which shows the effectiveness of this method of extraction in terms of enhancing the oxidative stability of flaxseed oil. As flaxseed oil has a higher level of unsaturation due to higher content of linolenic acid (18:3), it has low oxidative stability compared with other vegetable oils such as rapeseed oil (11.4 h) and soybean oil (10.2 h) and is similar to sunflower seed oil (7.1 h) (Kowalski et al., 2004). However, using olive leaves in the oil extraction could increase the induction period comparable with the rapeseed and soybean oils. There have been reports on the effect of olive leaves on the improvement of induction period of sunflower and sesame oils, which were extracted via simultaneous extraction by pressurized propane (Jaski et al., 2022, 2023). There are also reports that using rosemary and green tea incorporated with oilseeds while oil extraction by cold press could increase the oxidative stability of extracted oils (Ashrafi et al., 2023; Mikołajczak et al., 2021).

Results also showed that higher levels of leaves could decrease the induction period and stability of the extracted oils, which can be explained by the peroxidative effects of phenolic compounds at higher concentrations. There are previously published results on the peroxidative effects of phenolic compounds on higher concentration (Simić et al., 2007; Spiegel et al., 2023). Therefore, according to these results, there has to be some restriction in the level of olive leaves' incorporation with oilseeds during the oil extraction to avoid reaching an unsuitable amount of phenolic compounds. However, there should be further investigation on the type and amount of other minor compounds, particularly peroxidative elements such as Fe and Cu, which can also be the reason behind the oxidative effect of leaves at higher levels due to peroxidative component accumulation.

Performing a correlation between the obtained values of oxidative stability and other properties of the extracted oils showed that higher acid and peroxide values can decrease the oxidative stability. However, oxidative stability has positive correlation with total phenol and carotenoid contents (Figure 4).

Fatty acid composition and the ratio of fatty acids in vegetable oils are important from technological and nutritional points of view. Flaxseed oil is a rich source of linolenic acid (18:3), which is an essential oil belonging to the n‐3 group. Generally, there is a shortage in the daily diet in the level of n‐3 fatty acids compared to the n‐6 essential fatty acids, which can initiate and cause many disorders and diseases (Hashempour‐Baltork et al., 2018; Tang et al., 2021). Therefore, consuming and using vegetable oils rich in n‐3 fatty acids are necessary and recommended in the daily diet (Hashempour‐Baltork et al., 2022). On the other hand, vegetable oils such as flaxseeds with high content of unsaturated fatty acids are less stable and need unique preservation methods such as storage at the very low level of temperature and in dark condition and applying antioxidants.

Results showed that oil extracted from the flaxseeds had about 51%–57% linolenic fatty acid, followed by linoleic acid (14%–17%), oleic acid (14%–17%), palmitic acid (5%–8%), and stearic acid (3%–6%) (Table 3). The obtained results are in agreement with the many published data on the fatty acid composition of flaxseed oils (Calder & Yaqoob, 2009; Mikołajczak & Tańska, 2022). Generally, the incorporation of olive leaves with flaxseeds during the oil extraction did not affect the fatty acid composition of the extracted oils (Table 3).

**TABLE 3:** Changes in the fatty acid composition (%) of oil extracted by cold press from flaxseeds containing different levels of blanched olive leaves during storage.

Oil*	Fatty acids									
	C16:0		C18:0		C18:1		C18:2		C18:3	
	1**	90	1	90	1	90	1	90	1	90
Control	5.5^bA^ ***	7.8^aA^	3.1^bAB^	5.4^aA^	15.0^bA^	16.2^aAB^	17.1^aA^	15.0^bB^	55.9^aA^	51.2^bB^
Leaves without treatment										
2.5%	5.6^bA^	7.0^aA^	2.9^bAB^	5.9^aA^	15.4^bA^	16.0^aAB^	16.9^aA^	14.9^bB^C	56.3^aA^	51.5^bB^
5%	4.9^bA^	7.3^aA^	2.9^bAB^	4.0^aB^	14.8^bA^	16.5 ^aA^	17.4^aA^	15.3^bB^	57.0^aA^	51.8^bB^
7.5%	5.3^bA^	7.8^aA^	3.4^bA^	3.5^aB^	15.1^bA^	15.5^aB^	16.8^aA^	14.0^bC^	57.0^aA^	50.0^bB^
10%	5.2^bA^	8.2^aA^	2.9^bAB^	5.8^aA^	15.0^bA^	16.9^aA^	16.1^aA^	14.0^bC^	57.5^aA^	48.3^bC^
Leaves treated with hot water										
2.5%	5.7^bA^	5.9^aB^	3.4^bA^	6.0^aA^	14.8^bA^	15.9^aAB^	17.2^aA^	15.8^bAB^	57.1^aA^	52.4^bB^
5%	5.3^bA^	7.1^aAB^	2.8^bB^	5.1^aA^	15.1^bA^	16.2^aAB^	16.9^aA^	15.5^bAB^	56.9^aA^	53.0^bAB^
7.5%	5.0^bA^	6.2^aB^	2.9^bAB^	5.0^aA^	14.9^bA^	16.0^aAB^	17.4^aA^	16.2^bA^	57.7^aA^	54.2^bA^
10%	5.0^bA^	7.0^aAB^	3.1^bAB^	4.2^aB^	15.0^bA^	17.0^aA^	17.3^aA^	16.6^bA^	57.0^aA^	54.0^bA^

* Oil samples extracted from flaxseeds with different levels of olive leaves.

** Day of storage.

*** Different small letters in each row indicate a significant difference for each fatty acid between days of storage. Different capital letters in each column indicate significant differences for treatments. Generally, percent coefficient of variation (CV%) was less than 10.

The obtained results showed that there were significant changes in fatty acid composition after 90 days of storage at room condition (Table 3). Polyunsaturated fatty acids (18:2 and 18:3) were decreased due to oxidation as the PV increased during storage also proved these results. However, the obtained results showed that the rate of polyunsaturated fatty acid oxidation was less in the oil extracted from the flaxseeds with leaves, particularly blanched leaves, at a level of 5%–7.5% compared with the control sample (Table 3). It has been reported that flaxseed oil is sensitive to oxidation, and its 18:3 and 18:2 content has been decreased during processing and the storage (Simsek et al., 2015). The obtained results in this study agree with the previously published data (Jung et al., 2021; Mikołajczak & Tańska, 2022).

## 4 CONCLUSION

Flaxseed oil is a rich source of ω_3_ fatty acids, which makes it very valuable in the daily diet. Still, there is a limitation in its marketing due to its low oxidative stability. Incorporation of blanched olive leaves with flaxseeds while at cold press could produce an oil with higher bioactive components and oxidative stability compared to the oil extracted from flaxseeds without incorporation of olive leaves. However, there was an upper limit to the olive leaves (<7.5%) to use with flaxseeds, as in the higher level, it could reduce the oxidative stability and give a higher peroxide value. Also, there is a need for further detailed research to determine the leached bioactive components from the olive leaves to the extracted oils, and their nutritional effects and their changes during the storage and thermal processing.

## AUTHOR CONTRIBUTIONS

**Ramin Teimouri Okhchlar**: Methodology, Formal analysis, Investigation, Writing original draft, **Afshin Javadi**: Methodology, Supervision, Writing – review & editing, **Sodeif Azadmard‐Damirchi**: Conceptualization, Supervision, Methodology, Validation, Project administration, Writing – review & editing, **Mohammadali Torbati**: Visualization, Project administration, Writing – review & editing.

## FUNDING INFORMATION

There was no funding source to report.

## CONFLICT OF INTEREST STATEMENT

The authors have declared no conflict of interest.

## ETHICS STATEMENT

This study does not involve any human or animal testing.

## Data Availability

Data available on request from the authors.
